# Association between increased neutrophil-to-lymphocyte ratio and postoperative delirium in elderly patients with total hip arthroplasty for hip fracture

**DOI:** 10.1186/s12888-020-02908-2

**Published:** 2020-10-07

**Authors:** Rui He, Fei Wang, Huarui Shen, Yong Zeng, LijuanZhang

**Affiliations:** 1 Department of Joint Surgery, The Second People’s Hospital of Chengdu, Chengdu, 610021 People’s Republic of China; 2 Department of Joint Surgery, The Third People’s Hospital of Chengdu, Chengdu, 610000 People’s Republic of China; 3 Department of Joint Surgery, Affiliated Traditional Chinese Medical Hospital of Southwest Medical University of Sichuan Province, Luzhou, 646000 People’s Republic of China; 4 Department of Neurology, The Second Affiliated Hospital of Chengdu College, Nuclear Industry 416 Hospital, Chengdu, 610051 People’s Republic of China

**Keywords:** Total hip arthroplasty, Neutrophil-to-lymphocyte ratio, Postoperative delirium, Elderly patients

## Abstract

**Background:**

Delirium is a common complication in elderly patients with total hip arthroplasty (THA) for hip fracture. The mechanism of postoperative delirium (POD) is associated with the neuroinflammatory process. The aim of this study was to the incidence and perioperative risk factors of POD and investigate whether NLR could serve as a potential marker for POD in elderly patients with THA for hip fracture.

**Methods:**

This was a multicenter prospective study, we included elderly patients with THA for hip fracture under general anesthesia. Receiver operating characteristic (ROC) curve was performed to identify the optimal cut point of NLR for POD. The relationship between NLR and POD was analyzed by multivariable analysis.

**Results:**

Seven hundred eighty patients (mean age 73.33 ± 7.66) were eligible for inclusion in the study. 23.33% (182/780) of patients had POD. ROC curve analysis showed that the optimal cut point of NLR for POD was NLR ≥ 3.5. Compared with no POD, higher NLR, older age, diabetes, and higher neutrophil count were more likely in patients with POD(*P* < 0.05). Multivariate logistic regression analysis showed that NLR ≥ 3.50 [adjusted odds ratio(aOR), 3.93; confidence interval (CI), 2.47–6.25; *P* < 0.001)], older age (aOR, 1.04; 95%CI, 1.02–1.07; *P* = 0.001), diabetes (aOR, 1.58; 95% CI, 1.06–2.36; *P* = 0.025),higher neutrophil count (aOR, 1.25; 95%CI, 1.15–1.35; *P* < 0.001) were associated with increased risk of POD.

**Conclusions:**

Older age, diabetes, higher neutrophil count, and NLR ≥ 3.5 were independent risk factors for POD, and NLR can be used as a potential marker for prediction of delirium in elderly patients with THA for hip fracture.

## Background

Delirium is defined as an acute disorder of attention and cognition, it is a common complication of surgery, especially in elderly patients. Postoperative delirium (POD) is a well-described medical problem with long-term consequences of increased mortality and morbidity [1]. POD usually occurs within 5 days after surgery [2, 3]. Studies have shown that the incidence of POD is between 15.7–48% in elderly patients with hip fractures [4–6]. POD has a negative effect on postoperative recovery, increases the nursing burden of caregivers, extends the length of hospital stay, increases hospitalization costs and hospitalization mortality, and may even lead to long-term cognitive impairment [7–9]. Its pathogenesis is complex and remains to be clarified.

The pathophysiology of delirium is not fully understood, many disorders of physiological mechanisms may be involved, including neuroinflammation and the effects of oxidative stress [10, 11]. Considering delirium may be related to the pathological mechanism of inflammation, it is particularly important to find predictive markers in inflammatory factors. The scientific studies showed that certain inflammatory markers are associated with POD [12, 13], but they may be excluded in clinical practice due to cost or cumbersome diagnostic procedures. There are relatively few studies devoted to association serum biomarkers with POD [12, 14].

Neutrophil-lymphocyte ratio (NLR) is a marker of inflammation and oxidative stress. Previous study reported that NLR increased in elderly patients with delirium [15]. NLR had been used as a marker of inflammation in cardiovascular disease and diabetes [16, 17]. A study showed that NLR in peripheral blood can also be used as a simple systemic inflammatory response (SIR) marker and has diagnostic value for some diseases [18]. NLR has been shown to be a good predictor of outcomes for neurological and psychiatric disorders [19–21].

Increased NLR for developing delirium after total hip arthroplasty (THA) in elderly hip fracture patients have not been investigated. We hypothesized that in the elderly hip fracture patients with THA who had POD would be predicted by higher NLR value. Therefore, the current study was undertaken to investigate the incidence and perioperative risk factors of POD and investigate whether NLR could serve as a potential marker for POD in elderly patients with THA for hip fracture.

## Methods

### Study population

This study was a prospective multicenter study conducted in three medical centers: The Second People’s Hospital of Chengdu, The Third People’s Hospital of Chengdu, Affiliated Traditional Chinese Medical Hospital of Southwest Medical University of Sichuan Province. The study enrolled elderly patients with THA for hip fracture between March 2014 and December 2019 (registration number: ChiCTR2000037945). Patients were included in the study only if they fulfilled all the following criteria: 1. Aged ≥65 years; 2. Patients underwent the first THA procedure; 3. American Society of Anesthesiologists physical status I–III. The exclusion criteria were the following: 1.Owning infection at admission; 2 Postoperative infections; 3.A history of dementia or Mini-Mental State Examination (MMSE) score < 24; 4. Patients who underwent other surgery within 6 months; 5.A history of mental illness; 6.Cardiac (include acute myocardial infarction, congestive heart failure, a history of tachyarrhythmia/bradyarrhythmia or atrial fibrillation), pulmonary disease and impaired renal function (estimated glomerular filtration rate < 60 mL/min per 1.73 m^2^); 7.Using antipsychotic medications; 8.Alcohol or drug abuse; 9.Incapable of appropriate communication;10.Preoperative delirium, stroke after surgery and in-hospital mortality.

### Data collection

We collected all patients’ the baseline characteristics within 24 h after admission and before surgery. Routine laboratory tests were undertaken within 24 h after admission, including blood counts, blood chemistries, hepatic and renal function, blood glucose. The complete blood cell counts were analyzed using a Sysmex XE-5000 instrument (Sysmex, Kobe, Japan). The neutrophil count divided by the lymphocyte count yielded the NLR. CRP was detected with immunoturbidimetric method (Cobas 8000 c701/702 analyzer, Roche Diagnostics, Rotkreuz, Switzerland).

The symptoms and signs of infections were assessed, white blood cell count, procalcitonin (PCT), urine screening, chest CT, and body temperature (at least 3 times a day) were monitored within 7 days after admission. Infections included pulmonary infection, urinary tract infection and other localization’s infections were excluded.

To assess mental status, we used the Mini-Mental State Examination (MMSE) as an estimate of preoperative cognitive decline. A score lower than 24 on this scale indicates cognitive decline. Prefracture activities of daily living (ADL) functioning was assessed with the Barthel Index (BI).

Two research nurses trained in delirium assessment and not involved in the clinical care of participants performed all clinical assessments. The diagnostic criteria of delirium are described by the Diagnostic and Statistical Manual IV diagnostic criteria [22] and the Confusion Assessment Method (CAM) [23]. Delirium assessment contains four evaluation features: (i) acute change or fluctuating course of mental status; (ii) inattention; (iii) altered level of consciousness; (iv) disorganized thinking; A positive diagnosis of delirium required the presence of items (i) and (ii), and either (iii) or (iv). All patients were assessed for delirium once daily, using the CAM from time of admission to the seventh postoperative day. CAM was applied by two research nurses, based on a 10–30-min interview with the patient, together with information from close relatives, nurses, and hospital records. The CAM has been validated in several languages and replicated in multiple settings.

### Statistical analysis

Patients were classified into no POD and POD groups. The data are presented as numbers (%) or means (±standard deviations). Receiver operating characteristic (ROC) curve was used to determine the optimal NLR for POD. To identify differences between no POD and POD groups, the Pearson χ^2^ test was used for categorical variables. Student’s t-test was used to compare normally distributed variables. Mann-Whitney U tests were used to compare nonnormally distributed variables. Multivariate logistic regression analysis was performed to identify determinants independently associated with POD. Variables associated with POD in the univariate analyses with a *P*-value < 0.20 were included in the multivariate analysis. The results are expressed as the adjusted odds ratio (aOR) with their corresponding 95% confidence interval (CI). The data were analyzed using SPSS 22 software. *P* < 0.05 was considered statistically significant.

## Results

### Characteristics of the study subjects

**Fig. 1:**
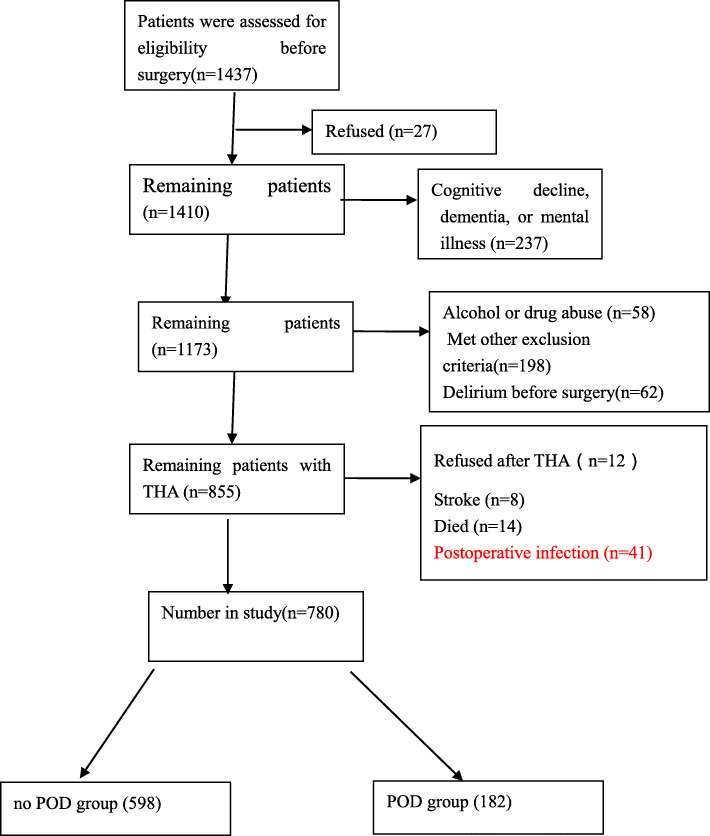
Patient’s flowchart

The patients were included for this study from three centers. A total of 1437 patients(≥65 years) with hip fracture who may undergo THA were included, 27 patients refused to participate in the study before THA, 237 patients had cognitive decline, dementia, or mental illness,58 patients had history of alcohol or drug abuse, and 198 patients were excluded because they met 1 or more of the other exclusion criteria, 62 patients had delirium before surgery. The remaining 855 patients, among them after THA,12 patients refused to participate in the study, 8 patients had stroke,14 had died, and 41 had infection, the remaining 780 patients were enrolled (Fig. 1), the mean age was 73.33 ± 7.66 years (65–97 years), comprised 48.59% (379) men. In the study population, 527 patients had a history of hypertension, 280 had a history of diabetes, 468 had a history of hyperlipidemia, 214 patients current Smoking. The median Barthel score was 89 points prior hip fracture. The mean Surgery duration was 87.31 min.

182(23.33%) patients experienced POD, the median hours between surgery and diagnosis of POD was 47.1 (range: 25.7–75.9). Baseline characteristics of patients in the no POD and POD groups were compared (Table 1). At baseline, patients with POD showed significantly older age (75.77 ± 8.57 vs73.28 ± 7.44, *P* < 0.001), higher NLR value [4.67 ± 1.62 vs 2.68 ± 1.26, *P* < 0.001], higher neutrophil count (6.67 ± 3.55 vs 3.77 ± 2.29,*P* < 0.001), higher percentage of diabetes [79(43.41%) vs201(33.61%), *P* < 0.001] than patients with no POD.

**Table 1:** Comparison of baseline characteristics between patients with no POD and POD groups

	no POD group (598)	POD group (182)	OR(95%CI)	P*
Age, y (Mean SD)	73.28 ± 7.44	75.77 ± 8.57		**< 0.001**
Female, n(%)	302 (53.40)	99 (46.39)	1.17 (0.84–1.63)	0.357
Male, n(%)	296 (46.60)	83 (53.61)	1.17 (0.84–1.63)	0.357
BMI ≥ 24 kg/m, n(%)	181 (28.40)	51 (31.96)	0.90 (0.62–1.30)	0.562
Current Smoking, n(%)	168 (25.24)	46 (30.41)	0.87 (0.59–1.27)	0.455
Hypertension, n(%)	404 (66.26)	123 (60.82)	1.00 (0.70–1.43)	0.995
Diabetes, n(%)	201 (33.61)	79 (43.41)	1.52 (1.08–2.13)	**0.016**
Hyperlipidemia, n(%)	364 (57.52)	104 (50.00)	0.86 (0.61–1. 20)	0.369
Duration of fracture, d (Mean SD)	10.29 ± 11.09	9.23 ± 10.19		0.302
Admission to operation duration, h (Mean SD)	61.14 ± 21.59	59.14 ± 21.16		0.283
Surgery duration, (Mean SD)	79.87 ± 18.03	82.56 ± 18.14		0.077
Anesthesia duration, h (Mean SD)	99.53 ± 18.41	102.23 ± 18.15		0.088
Barthel score, (Mean SD)	87.475 ± 12.20	88.90 ± 12.32		0.167
MMSE score, (Mean SD)	27.63 ± 1.75	27.36 ± 1.73		0.068
Neutrophil count (×10^9^/L), (Mean SD)	3.77 ± 2.29	6.67 ± 3.55		**< 0.001**
Lymphocyte count (× 10^9^/L), (Mean SD)	1.40 ± 0.51	1.40 ± 0.58		0.917
CRP (mg/L), (Mean SD)	1.35 ± 0.50	1.38 ± 0.54		0.596
Glucose (mg/dL), (Mean SD)	136.99 ± 22.54	138.97 ± 22.72		0.402
NLR value, (Mean SD)	2.68 ± 1.26	4.67 ± 1.62		**< 0.001**
NLR ≥ 3.50, n(%)	156 (26.09)	136 (74.73)	8.38 (5.72–12.26)	**< 0.001**

Bold indicates *P*-values less than 0.05

*Comparison between no POD and POD groups. The data are presented as numbers (%) or mean values (±standard deviation). The Pearson χ^2^ test was used for categorical variables. Student’s t-test was used to compare normally distributed variables. Mann-Whitney U tests were used to compare nonnormally distributed variables

### Evaluation of the prognostic value of NLR for POD

**Fig. 2:**
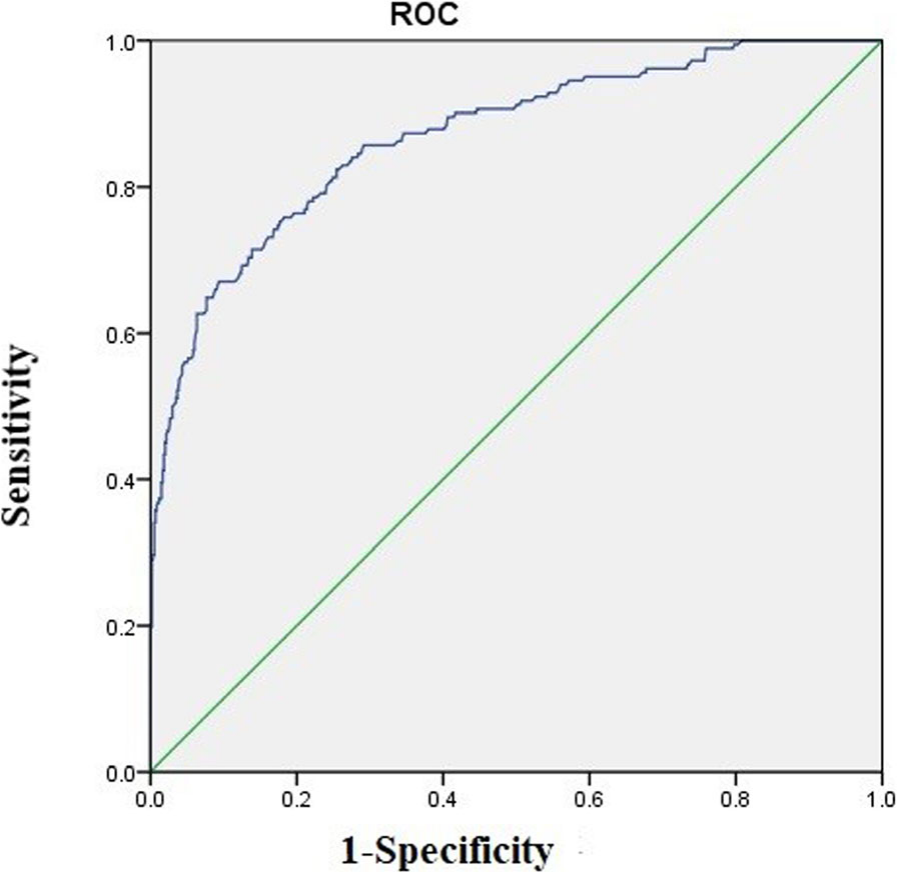
Receiver operating characteristic curve analysis for NLR for prognostic value for POD

ROC curve analysis showed high accuracy for NLR to predict POD with AUC of 0.83 (95% CI 0.80 to 0.86) (Fig. 2). Using a cut off value for NLR ≥ 3.50, the sensitivity was 74.73%, and the specificity was 73.91%, providing a positive predictive value (PPV) for POD of 46.57%. Compared with patients no POD, those with POD had higher prevalence of NLR ≥ 3.50(74.73% vs 26.09%,*P* < 0.001).

### Multivariable models on the association between NLR ≥ 3.50 and POD

Unadjusted logistic regression analysis identified older age (OR,1.04; 95%CI,1.02–1.07; *P* < 0.001), percentage of diabetes (OR,1.52; 95%CI,1.08–2.13; *P* = 0.016), higher neutrophil count (OR,1.43; 95%CI,1.33–1.53; *P* < 0.001), and higher NLR value (OR,2.78; 95%CI,2.35–3.29; *P* < 0.001) as factors associated with a risk predictor of POD. When the factors associated with POD in the univariate analyses (*P* < 0.20) were entered into the multivariate logistic regression analysis (adjusted for age, Barthel score, NLR value or NLR ≥ 3.50, neutrophil count, surgery duration, anesthesia duration, MMSE score, diabetes), the results showed that older age (aOR, 1.05; 95% CI, 1.02–1.07; *P* = 0.001), higher NLR value (aOR, 2.74; 95% CI, 2.20–3.42; *P* < 0.001), higher neutrophil count (aOR, 1.25; 95% CI, 1.15–1.35; *P* < 0.001),and diabetes (aOR, 1.69; 95% CI, 1.11–2.58; *P* = 0.015) were associated with increased a risk of POD (Table 2). When NLR ≥ 3.50 was entered into multivariate logistic regression (Model 2), NLR ≥ 3.50 resulted to be associated with increased risk POD (aOR, 3.93; 95%CI, 2.47–6.25; *P* < 0.001), older age (aOR, 1.04; 95%CI, 1.02–1.07; *P* = 0.001),diabetes (aOR, 1.58; 95% CI, 1.06–2.36; *P* = 0.025) were associated with increased risk POD (Table 2).

**Table 2:** Multivariable models showing factors associated with POD

	OR (95% CI)	P*
Model 1(NLR value)		
Older age	1.05 (1.02–1.07)	**0.001**
Higher NLR value	2.74 (2.20–3.42)	**< 0.001**
Diabetes	1.69 (1.11–2.58)	**0.015**
Model 2(NLR ≥ 3.50)		
Older age	1.04 (1.02–1.07)	**0.001**
Neutrophil count	1.25 (1.15–1.35)	**< 0.001**
NLR ≥ 3.50	3.93 (2.47–6.25)	**< 0.001**
Diabetes	1.58 (1.06–2.36)	**0.025**

Bold indicates *P*-values less than 0.05

*Multivariable adjusted for age, Barthel score, NLR value (NLR ≥ 3.50), neutrophil count, surgery duration, anesthesia duration, MMSE score, diabetes

## Discussion

In this study, we investigated incidence and perioperative risk factors of POD in order to determine whether pre-existing conditions and NLR would predict the risk of POD. In this study, we found a high incidence of POD in the elderly patients undergoing hip fractures surgery, 182 (23.33%) elderly patients experienced POD after THA, which was consistent with previous studies [24–26]. ROC curve analysis showed that NLR ≥ 3.5 was a useful marker for predicting POD, the AUC of 0.83 in POD group further indicated that NLR was predictive of POD with high specificity. We identified risk factors associated with POD. Compared with patients without POD, those with POD were more likely to had older age, diabetes, higher NLR value, higher neutrophil count. Multivariate logistic regression analyses indicated that older age, higher NLR value, diabetes were independent risk factors of POD in elderly patients after THA for hip fracture, elderly patients with NLR ≥ 3.5 after THA had about 4-fold increased risk of POD.

Delirium is a common and serious complication in elderly patients after surgery because it can lead to decreased function and longer hospital stays [27–30]. Patients with delirium during hospitalization had a higher 6-month mortality rate than those without delirium [31, 32]. Increased morbidity and mortality, longer hospital stays, cognitive and functional decline, and increased institutionalization are all likely to increase social and health care costs [31, 32]. Our findings have important implications for clinical prevention and early treatment of delirium, NLR is easy to obtain in clinical practice, it is cost-effective.

In the community, the prevalence of delirium among normal elderly people is low (1–2%), but increases with age, the prevalence increases to 14% in older people who over 85 years [33, 34]. The prevalence of delirium is 10–30% in older adults who visit the emergency department [35]. The prevalence of delirium is highest among older adults hospitalized. Previous studies have shown that the prevalence of delirium in older patients in the ICU is significantly higher than in younger patients, this finding supports the notion that older age is an important risk factor for delirium [36].

The mechanism of the relationship between age and the POD may be that the ability to respond to stress and adapt to abnormal metabolism was reduced in older patients, and this dysfunction was accompanied by decreased brain volume and decreased physical activity [37]. In addition, with the increase of age, the disorder of various neurotransmitter systems may be the main pathological cause of POD [38].

In this study, we found that diabetes was associated with about 1.6-fold higher risk of POD. Possible causes of POD include cerebral perfusion injury during surgery [39], persistent neurotoxic effects after anesthesia, and perioperative opioid use, all of which have negative effect on brain function [40, 41]. Diabetic patients generally present with brain microvascular lesions [42, 43] and large vascular injury [44]. Compared with non-diabetic patients, the risk of delirium is increased in diabetic patients [45, 46]. A previous observational study found that surgery in diabetic patients may accelerate the negative effects of high blood sugar on brain function. Other factors associated with delirium include an up-regulation of sympathetic tone and a down-regulation of parasympathetic tone due to surgical stress, impaired cholinergic function, reversible damage to brain oxidative metabolism, abnormal neurotransmitter pathways, and the release of neuroinflammation [47]. Hyperglycemia is a known factor inducing neuroinflammation and a physiological response to inflammation and is also an important cause of delirium in diabetic patients.

Under pathological conditions, the relationship between NLR and delirium has not been clarified. A recent study had found that NLR can be a predictive factor for delirium after acute ischemic stroke [48]. The data regarding the NLR in POD had not been studied previously. In the present study, we found that increased NLR was an independent risk factor for developing POD in hip fracture patients with THA. The NLR was elevated in POD group (4.67 ± 1.62 vs. 2.68 ± 1.26, *P* < 0.001), yet AUC for NLR was 0.83, indicating higher predictive ability. Even at the optimal cut-off of 3.50, the sensitivity was 74.73% and specificity was 73.91%, making it an ideal test for the detection of POD in elderly patient with THA for hip fracture. This study showed that higher NLR value was associated with a 2.74-fold higher risk of POD, when NLR ≥ 3.5 used for predicting POD, we found that NLR ≥ 3.5 could be an independent risk factor for POD. Our investigation added an important evidence on the relationship between delirium and a dysfunction of immune effector cells. This study found that the immune system and oxidative stress may play an important role in the pathogenesis of POD. Previous studies have shown that inflammatory markers and cytokines can be detected in the cerebrospinal fluid of patients with delirium [49–51]. A growing evidence suggests that neutrophils and lymphocytes are major contributors to acute inflammation [52]. The generalized stress can lead to nonspecific activation of the immune system, increasing the neutrophil count and decreasing the lymphocyte count [52, 53]. Neutrophils were once activated, they release reactive oxygen species, myeloperoxidase, and proteolytic enzymes [53, 54], which disrupted the blood–brain barrier (BBB) and damaged brain [55]. Decreased lymphocytes, increased levels of catecholamine and cortisol, redistribution of lymphocytes to lymphatic tissue, and accelerated apoptosis, combined the stress of the surgery might lead to a detrimental inflammatory state, which might be underlying mechanisms for delirium [55, 56].

Some limitations of this study are worth considering. First, we relied on a single baseline NLR value, lack of dynamic change of NLR, which may provide additional information about development for delirium. Second, we lacked data on APACHE scores, post-operative features that may have affected the outcomes we studied, such as plasma albumin levels, hemoglobin levels, duration spent in intensive care unit, care level, postoperative rehabilitation. Third, we did not evaluate the duration of delirium. Fourth, we did not distinguish between hyperactive versus hypoactive delirium. Fifth, patients with dementia or MMSE score < 24 were excluded, which limits the generalizability of the results. Despite these limitations, our study is a multicenter clinical study, included the large number of patients, the collection of several important clinical data, the adjustment of data analyses for a wide variety of confounding factors. In addition, it is the first study to evaluate the association between NLR and POD among elderly patients with THA for hip fracture.

## Conclusions

In conclusion, we evaluated found that older age, diabetes, and NLR value were independent risk factors for POD in elderly patients with THA for hip fractures, and NLR can be used as a potential marker for prediction of POD after hip surgery.

## Data Availability

The datasets used and/or analyzed during the current study are available from the corresponding author on reasonable request.

## Abbreviations

CI: Confidence interval
M: Mean
OR: Odds ratio
SD: Standard deviation
POD: Postoperative delirium
NLR: Neutrophil-to-lymphocyte ratio
THA: Total hip arthroplasty
